# Deep learning prediction of two-dimensional ocean dynamics with wavelet-compressed data

**DOI:** 10.3389/frai.2022.923932

**Published:** 2022-10-21

**Authors:** Ali Muhamed Ali, Hanqi Zhuang, Ali K. Ibrahim, Justin L. Wang, Laurent M. Chérubin

**Affiliations:** ^1^Harbor Branch Oceanographic Institute, Florida Atlantic University, Boca Raton, FL, United States; ^2^Department of CEECS, Florida Atlantic University, Boca Raton, FL, United States; ^3^Department of Computer Science, University of Illinois Urbana Champaign, Champaign, IL, United States; ^4^Harbor Branch Oceanographic Institute, Florida Atlantic University, Fort Pierce, FL, United States

**Keywords:** sea surface height, loop current forecast, long short term memory, empirical orthogonal function, wavelet transform, deep learning

## Abstract

This study addresses the challenge represented by the application of deep learning models to the prediction of ocean dynamics using datasets over a large region or with high spatial or temporal resolution In a previous study by the authors of this article, they showed that such a challenge could be met by using a divide and conquer approach. The domain was in fact split into multiple sub-regions, which were small enough to be predicted individually and in parallel with each other by a deep learning model. At each time step of the prediction process, the sub-model solutions would be merged at the boundary of each sub-region to remove discontinuities between consecutive domains in order to predict the evolution of the full domain. This approach led to the growth of non-dynamical errors that decreased the prediction skill of our model. In the study herein, we show that wavelets can be used to compress the data and reduce its dimension. Each compression level reduces by a factor of two the horizontal resolution of the dataset. We show that despite the loss of information, a level 3 compression produces an improved prediction of the ocean two-dimensional data in comparison to the divide and conquer approach. Our method is evaluated on the prediction of the sea surface height of the most energetic feature of the Gulf of Mexico, namely the Loop Current.

## 1. Introduction

Today's full-water column and seas surface height (SSH) predictions primarily rely on the use of finite-difference, finite-volume, and finite-element methods to solve the primitive equation of motion in numerical models used to simulate ocean dynamics. The outputs of these models consist of the temporal prediction of three or two-dimensional fields of ocean state variables including the velocity field, temperature, salinity, and SSH, which expresses the complex subsurface dynamics of the ocean. Since the 1990s, the field of machine learning has provided improved methods to retrieve two-dimensional information from satellite measurements, such as surface wind speed (Krasnopolsky et al., 1995), long wave net radiation at the sea surface (Liu et al., 1997) and ocean surface specific humidity and air temperature (Jones et al., 1999). Machine-learning-based approaches benefit from their ability to represent both nonlinear and stochastic phenomena (Zhang et al., 2017; Song et al., 2020).

Deep learning is a specific machine-learning method that was developed on the basis of imitating the neural structure of information processing of the human brain to extract features from input data, enabling a machine to understand the underlying information in the data and obtain specific information (Xiao et al., 2020). Particularly, as one of the deep learning models, the Recurrent Neural Networks (RNN) (Elman, 1990) was designed to extract dynamic time series and temporal features through the context of events, which forms the basis of predictions. However, RNNs suffer from the gradient vanishing problem making them unable to adjust or learn from long-term dependencies (Bengio et al., 1994; Schaefer et al., 2008; Pascanu et al., 2013).

To address this problem (Hochreiter and Schmidhuber, 1997) proposed the Long Short-Term Memory (LSTM) network in the late 1990s which has been widely used in many fields, including the short- and mid-term predictions of oceanic features (Zhang et al., 2017; Liu et al., 2018; Wang et al., 2019; Xiao et al., 2019; Muhamed Ali et al., 2021). LSTM networks have outperformed fully connected neural networks and other machine learning techniques in natural language processing (Salehinejad et al., 2017; Al-Rfou et al., 2019) that has many similarities with ocean current predictions, as shown by Immas et al. (2021). In addition, this type of network has seen an increase in real-life applications, including but not limited to aquaculture (Banan et al., 2020), wind and solar energy resources management (Shamshirband et al., 2019), and also in industrial applications (Fan et al., 2020).

In a recent study by Wang et al. (2019), an LSTM model was used for the prediction of the SSH of the most energetic circulation feature of the Gulf of Mexico (GoM), namely the Loop Current. It is a strong pulsating current that forms a circulation loop in the eastern Gulf of Mexico and that sheds large anticyclonic eddies at irregular intervals ranging from 3 to 18 months (Chérubin et al., 2005, 2006; Donohue et al., 2016). The dataset used in Wang et al. (2019) consisted of 18 years of simulated SSH at 1/25^*o*^ horizontal resolution for the entire GoM. To cope with the data density, Wang et al. (2019) chose to split the computational domain into smaller non-overlapping sub-domains. This approach called the “divide and conquer” (DAC) method led to the implementation of an LSTM model for each sub-domain. In order to ensure the continuity of the sub-domain solutions across their boundaries, a weighted smoothing algorithm was applied at each time step of the prediction process. Despite the smoothing of discontinuities, this approach leads to the growth of unrealistic SSH features in the predicted SSH. Using metrics set in the literature for LC prediction, the LSTM DAC method predicted the Loop Current System frontal distance from reference points within 40 km, 9 weeks in advance in advance vs. 4–5 weeks for ocean conventional numerical models (Oey et al., 2005; Wang et al., 2019). Furthermore, the model predicted the final separation of eddies Cameron and Darwin 8 and 12 weeks in advance, respectively.

In the study herein, the data size limitation is addressed by applying a data compression technique to reduce the size of the data while preserving the dynamical information relevant to the LC dynamics and its prediction. The compression method consists of the Discrete Wavelet Transform (DWT) used to conduct two-dimensional wavelet decomposition. Each two-dimensional data frame is decomposed into two components, a high frequency part, called “Detail” and a low frequency part called “Approximation” (Ergen, 2012). The resulting Approximation corresponds to a compressed version of the original data with half its resolution. This method is evaluated on the same dataset as in the Wang et al. (2019) study with the same computational constraints. The LSTM model is thus implemented on the entire domain of the compressed SSH data, which does not require partitioning to predict the future evolution of the SSH.

The remainder of the paper is organized as follows: Section 2 describes the different components of this new model including the DWT, the Empirical Orthogonal Function (EOF) decomposition, and the LSTM model, which is hereafter called the Wavelet-EOF-LSTM Learning (WELL) model. Then the region of interest, the data set, and the model performance metrics are also presented in Section 2. In Section 3 the WELL model SSH predictions are evaluated against the DAC model. Concluding remarks are given in Section 4.

## 2. Methods

**Figure 1:**
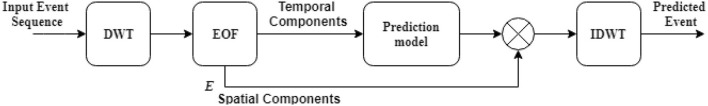
Block diagram of the proposed WELL model. DWT stands for Discrete Wavelet Transform, EOF for Empirical Orthogonal Function, and IDWT for Inverse DWT.

The WELL model consists of the following sequential blocks (Figure 1). First, the DWT is applied to each frame of the SSH tensor, which results in Approximation and Details. The Approximation series is further decomposed by an EOF into its temporal (principal components or PCs) and spatial components (modes). The evolution of PCs is used to train the LSTM model, which will then be used to predict their temporal evolution. The predicted SSH is obtained by applying the inverse EOF and DWT to the predicted PCs.

### 2.1. Wavelet decomposition

**Figure 2:**
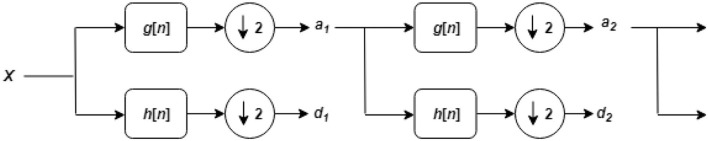
Two levels of Discrete Wavelet Transform. *g*[*n*] and *h*[*n*] denote low and high pass filters, respectively. Number 2 indicates that the output of each filter is decimated by a factor of two. *a*_*i*_ denote approximation coefficients and *d*_*i*_ detail coefficients.

The main benefit of DWT is its multi-resolution scale analysis ability (Mallat, 1999). The DWT either compresses the signal (high pass), which provides the detailed hidden information in the signal, or expands it (dilates, low pass) to provide approximate information. The transformed signals with high (detail) and low (approximation) frequencies (here wavenumbers) are analyzed independently. The approximate component is further transformed into sub-level detail and approximate signals which constitutes a multi-level decomposition (Figure 2) and reduces the dimensions of the input. The Approximation and Detail coefficients for each level can be calculated by using Equations (1) and (2).


(1) $$\begin{array}{l} y_{\text{low}}\left[n\right]=\overset{\infty }{\underset{k=-\infty }{\sum }}x\left[k\right]g\left[2n-k\right] \end{array}$$

(2) $$\begin{array}{l} y_{\text{high}}\left[n\right]=\overset{\infty }{\underset{k=-\infty }{\sum }}x\left[k\right]h\left[2n-k\right] \end{array}$$

where *x*[*k*] is the input signal; *g*[*n*] and *h*[*n*] are low and high pass filters, respectively; and *y*_*low*_ and *y*_*high*_ are the output of the low and high pass filters, respectively (Figure 2).

For each level of decomposition shown in Figure 2, *a*_*i*_ is used for the next step (or scale) transformation, and *d*_*i*_ is considered as high frequency noise in the data and, thus, not used. At scale *i*+1, the dimension of *a*_*i*+1_ and *d*_*i*+1_ is reduced by half from scale *i*. DWT reduction can continue until the dimension of *a*_*i*_ is reduced to two. In this study, the MATLAB Wavelet Toolbox DWT was applied using the order 4 Daubechies wavelet.

### 2.2. Empirical orthogonal function

The data used in this analysis consists of time series of spatial maps, such as SSH. A useful technique for compressing the variability of this type of time-series data is EOF, which is a form of principal component analysis (Thomson and Emery, 2014). The data is decomposed on orthogonal spatial modes, whose net response as a function of time accounts for the combined variance in all of the modes. However, there is no direct physical or mathematical relationship between the statistical EOFs and any related dynamical modes. The time-varying amplitude or PC of each orthogonal mode is obtained through the singular value decomposition (SVD) as shown in the following. Let's consider the following SVD equation:


(3) $$\begin{array}{l} X=UDW^{T} \end{array}$$

where *X* is an *n*x*p* array of spatial data points over time; *D* is an *n*x*p* rectangular diagonal matrix of non-negative values, the singular values of *X*; *U* is an *n*x*n* matrix, whose columns are orthonormal vectors of length *n*, called the left singular vectors of *X*; and *W* is a *p*x*p* matrix, whose columns are orthonormal vectors of length *p*, called the right singular vectors of *X*, which is the array of time varying SSH fields. *UD* contains the time-dependent PCs and *W*^*T*^ the stationary patterns or EOF modes.

Let *P* = *UD* and *E* = *W*^*T*^. Equation (3) can be written as:


(4) $$\begin{array}{l} X=PE \end{array}$$

and


(5) $$\begin{array}{l} \underset{X}{\underset{︸}{\left[\begin{array}{ccc} a_{1,1} & \ldots & a_{1,m}\\ \vdots & \ddots & \vdots \\ a_{n,1} & \ldots & a_{n,m} \end{array}\right]}}=\underset{PCs\;P}{\underset{︸}{\left[\begin{array}{ccc} p_{1,1} & \ldots & p_{1,m}\\ \vdots & \ddots & \vdots \\ p_{n,1} & \ldots & p_{n,m} \end{array}\right]}}\;\underset{EOFs\;E}{\underset{︸}{\left[\begin{array}{ccc} e_{1,1} & \ldots & e_{1,m}\\ \vdots & \ddots & \vdots \\ e_{m,1} & \ldots & e_{m,m} \end{array}\right]}} \end{array}$$

Let α_*i*_ be the diagonal elements of *D* with *i* = 1, 2, 3, .., *p*. The amount of variance *V*_*i*_ contained in the spatial pattern *i* with respect to the total variance can be calculated as follows:


(6) $$\begin{array}{l} V_{i}=\frac{\alpha_{i}^{2}}{\sum_{i=0}^{p}\alpha_{i}^{2}} \end{array}$$

It is shown here that the PCs, collectively, represent the total variance of the data field (Zeng et al., 2015; Hall and Leben, 2016).

### 2.3. Long short term memory network prediction sequence

The LSTM network used in this study is described in Muhamed Ali et al. (2018) and Wang et al. (2019). The MATLAB Neural Network Toolbox was used to implement the LSTM network. After balancing the computational cost and the accuracy performance, one shallow LSTM layer with 1, 800 hidden nodes was selected. The hyper-parameters were adjusted manually in a two-stage process. First the hyper-parameters were tuned by investigating their effect on the mean squared error of the SSH during the training stage, where the rate of convergence is used to identify the best learning curve of the LSTM network. In the second stage, the hyper-parameters were fine tuned by investigating the results in sliding windows to avoid overfitting. During this process, the number of hidden nodes and the number of epochs were found to be the most sensitive parameters to tune. The initial learning rate was chosen at 0.001, the gradient decay factor at 0.9, and the batch size at 64. The LSTM network was both trained and tested with SSH Approximation PC vectors. In the prediction phase, after each prediction, the LSTM updates its state in accordance with its own prediction. This allows the LSTM to continue predicting based on both the original training data and future predictions. In addition, new SSH observations can be added at any time to the training data set, which are then used to retrain the LSTM model.

### 2.4. SSH dataset and computational domain

The SSH dataset used in this study was obtained from the HYCOM + CFSR Gulf of Mexico Experiment (GOMl0.04/expt_02.2) provided by the HYCOM Consortium. The SSH data is preferred over the sea surface temperature (SST) because the former is the most affected by the Loop Current System, and it is the most accurate of all variables provided by HYCOM (Rosburg et al., 2016). The data used in this study spans from January 1992 to December 2009, containing a total of 6,574 days or approximately 954 weeks of SSH. In total, 38 LC eddies were formed during this period. The first 90% of the available data were used for training, and the remaining 10% for testing and validation. In order to perform weekly predictions, the daily SSH maps were decimated to weekly time series, i.e., one SSH map per week. The HYCOM SSH data is referred to the “observed” field in the rest of the study. The LSTM model was trained with weekly SSH fields from January 1992 to April 2008. The predictions were conducted for each week of the period March 2008 to December 2009 over 20-week windows each.

### 2.5. Model performance measures

**Figure 3:**
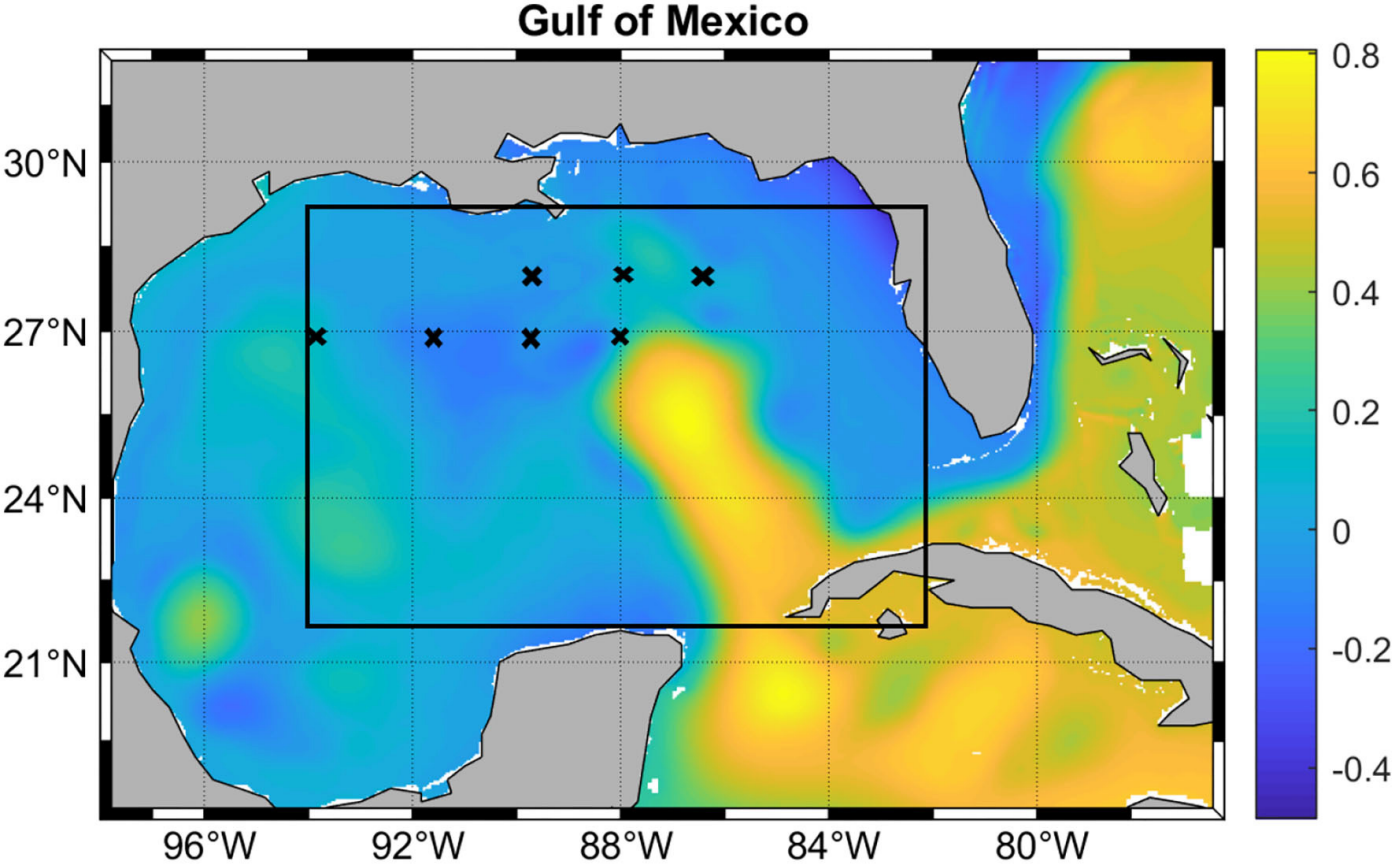
Model domain overlaid with SSH in meters. The black rectangle shows the region of interest (ROI) described in the text. The crosses mark the reference points used to measure the SSH prediction accuracy.

While the LSTM model can predict the SSH of the entire GoM, the evaluation of the predictions was focused on the Loop Current System in the region of interest (ROI) outlined in Figure 3. This is the region in which the LC is the most active and where eddy separation occurs and westward drift begins (Chérubin et al., 2005, 2006). It does not include land, which would have to be removed from the SSH matrix otherwise before reduction. Because HYCOM SSH was chosen, the SSH pattern sequences that the deep learning model has learned are intrinsic to the dynamics of the HYCOM model. Therefore, the skill measurement can only be done with HYCOM, which in this case represents the “true” ocean.

The same performance measures of the prediction skills as in Wang et al. (2019) were used in this study. The Correlation Coefficients (CCs) and Root Mean Square Errors (RMSEs) between “observed” and predicted fields were calculated. In order to evaluate the WELL model prediction against previous models, we used the same metric as in Oey et al. (2005), Zeng et al. (2015), and Wang et al. (2019). The Loop Current System frontal distances to 7 reference points, referred to as the Frontal Position Error (FPE) were also evaluated. As in Zeng et al. (2015), the front was defined by the 0.45 m contour line, and the same reference points as in Oey et al. (2005), Zeng et al. (2015), and Wang et al. (2019) were used (Figure 3). The Frontal position Root Mean Square Error (*FP*_*RMSE*_) is used to measure the accuracy of the predicted LC and LCE positions. This measure consists of the difference between the distance *d*_*p,r*_ from a predicted frontal position to a reference point and the distance *d*_*o,r*_ from the corresponding observed frontal position to the same reference point. *FP*_*RMSE*_ is averaged over all the reference points as follows:


(7) $$\begin{array}{l} FP_{RMSE}=\sqrt{\frac{\sum_{r=1}^{R}{\left(d_{p,r}-d_{o,r}\right)}^{2}}{R}} \end{array}$$

where *R* is the number of reference points used in the calculation. We also used a contour similarity measure such as the Modified Hausdorff Distance (MHD) (Hiester et al., 2016) to estimate the mismatch between curves. This measure exhibits a high sensitivity to outliers and can be expressed as follows. Given two corresponding sets of points, let


(8) $$\begin{array}{l} dis\left(A,B\right)=\frac{1}{\left|A\right|}\underset{a\in A}{\sum }dis\left(a,B\right);\;dis\left(a,B\right)=inf_{b\in B}\;dis\left(a,b\right), \end{array}$$

and


(9) $$\begin{array}{l} dis\left(B,A\right)=\frac{1}{\left|B\right|}\underset{b\in B}{\sum }dis\left(A,b\right);\;dis\left(A,b\right)=inf_{a\in A}\;dis\left(a,b\right), \end{array}$$

The MHD is then the maximum value between them:


(10) $$\begin{array}{l} MHD=max\left\{dis\left(A,B\right),dis\left(B,A\right)\right\} \end{array}$$

where *A* represents a set of sample points on the observed contour, *B* represents a set of sample points on the predicted contour, *dis*(*a, b*) is the Euclidean distance between point *a* and point *b*, and *dis*(*a, B*) is the minimum distance between *a* and each of the points in set *B*. Simply put, MHD is the larger of two averages, each of which is the average of the distances from individual sampled points on one contour to the other (Hiester et al., 2016).

## 3. WELL model prediction of the loop current system SSH

First, we address the effects of the DTW spatial compression process on the SSH input of the WELL model. Then the model prediction skills are assessed with the performance measures defined in Section 2.5. The assessment is focused on the prediction of eddies Cameron and Darwin life cycle and final separation, which are two LC eddies that were formed during the test period of this study, in July 2008 and February 2009, respectively. The WELL model is also evaluated against the SSH prediction of the DAC model by Wang et al. (2019).

### 3.1. Effects of DWT compression on SSH input

**Figure 4:**
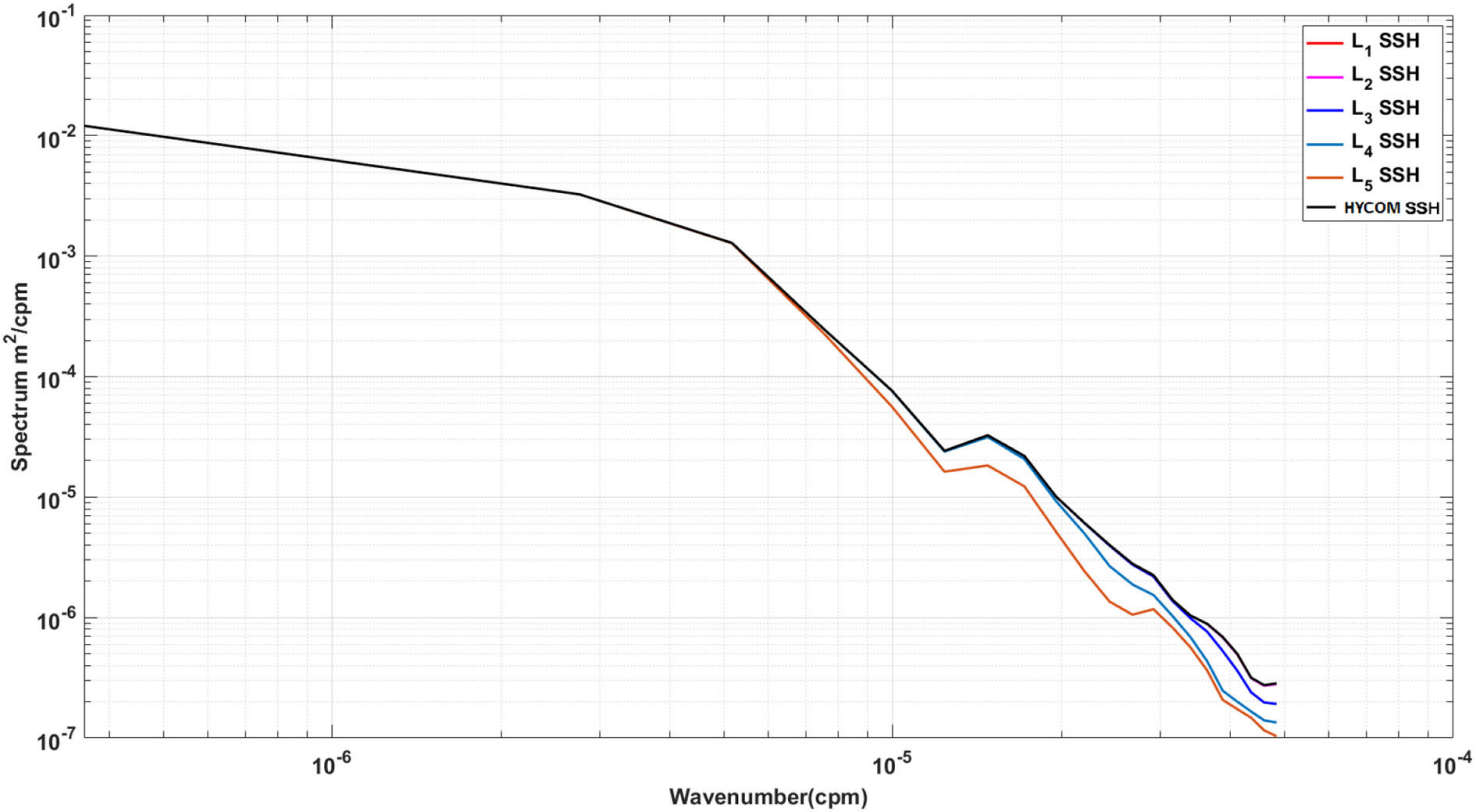
Five levels of DWT Approximations SSH spectra.

**Figure 5:**
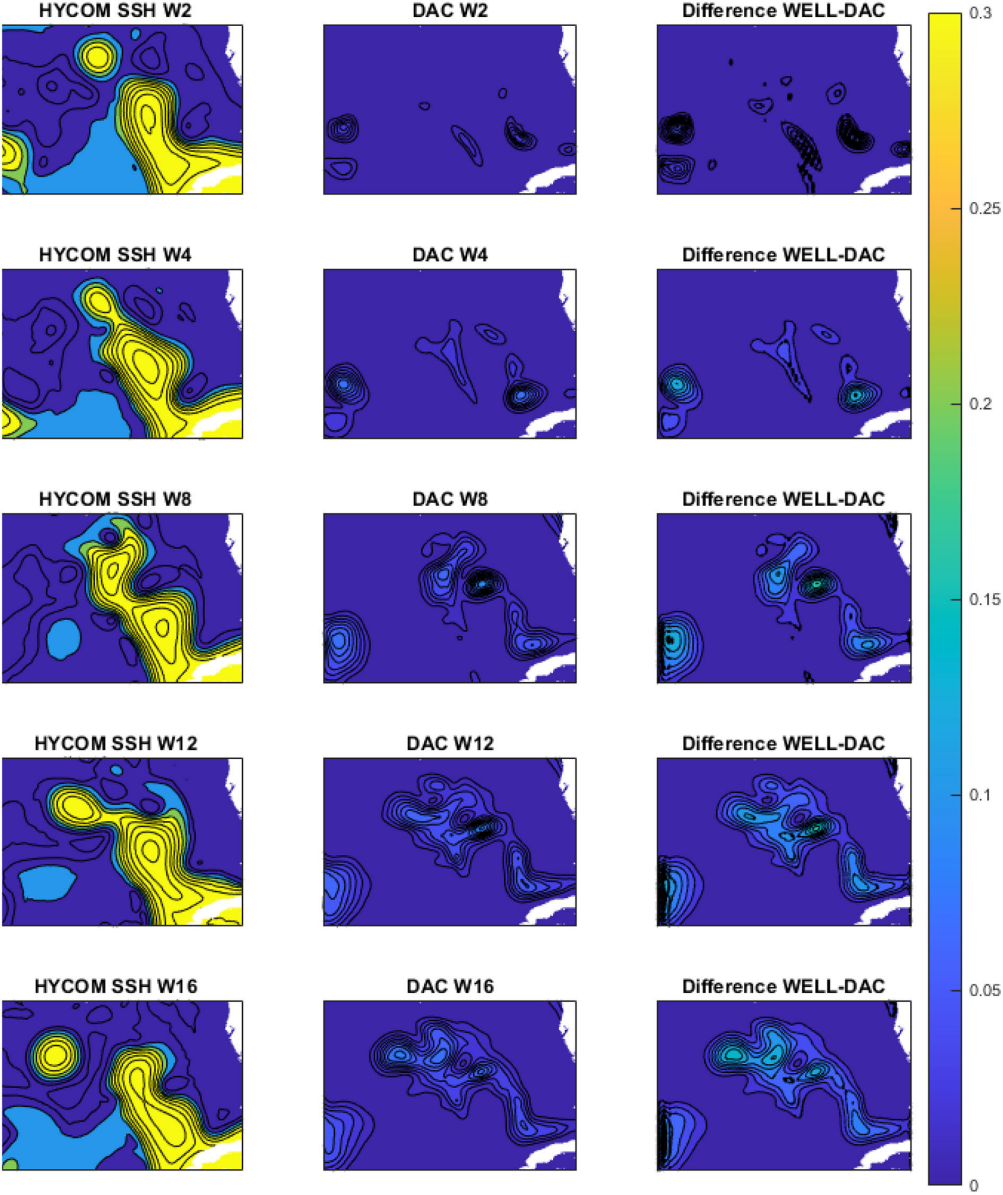
Seas surface height (m) and variance (*m*^2^) over the period November 1992 to March 1993, which is part of the training period. The left column shows the HYCOM SSH; the middle column shows the variance of the DAC model SSH; the right column shows the difference in variance between the WELL 3-level compressed SSH and the DAC model SSH. W2 - W16 indicates the number of weeks starting on the first week of November 1992.

**Figure 6:**
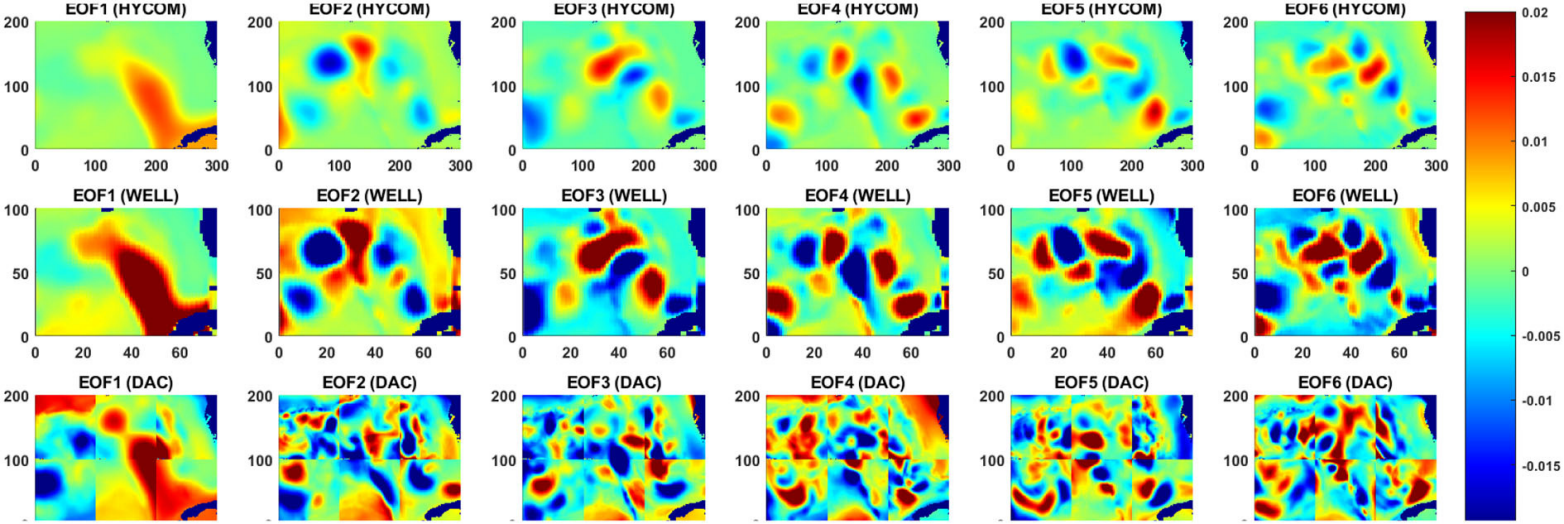
First 6 EOF modes for the original SSH **(top row)**, the 3-level compressed SSH **(middle row)**, and for the DAC model input SSH in which the EOF decomposition was applied to each sub-domain.

**Figure 7:**
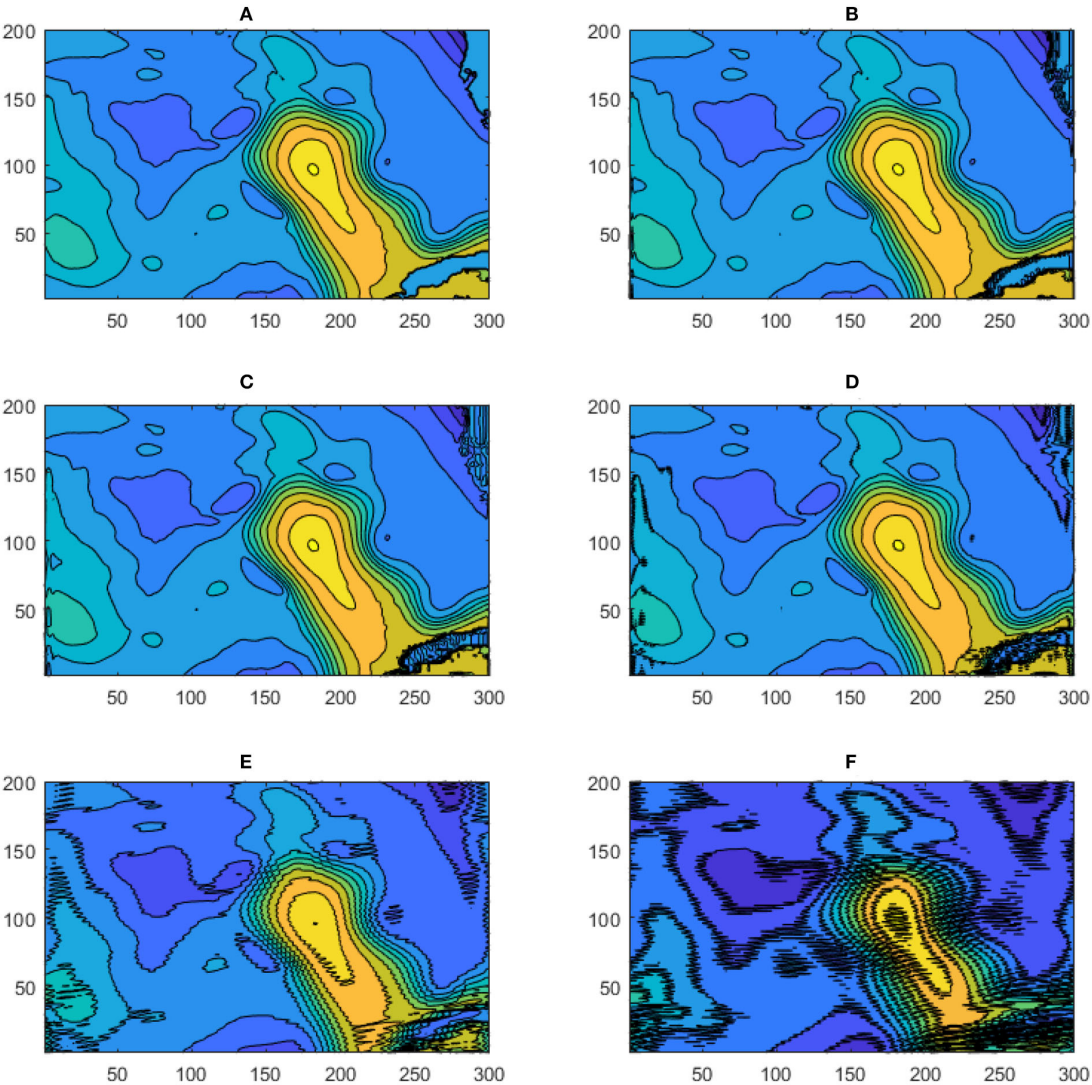
Reconstructed SSH for five DWT compression levels. **(A)** Observed SSH; **(B)** SSH reconstructed from the level 1 DWT compression; **(C)** SSH reconstructed from the level 2 DWT compression; **(D)** SSH reconstructed from the level 3 DWT compression; **(E)** SSH reconstructed from the level 4 DWT compression; **(F)** SSH reconstructed from the level 5 DWT compression. Fifteen contour levels from -50 to +100 cm with 10cm intervals are shown. The numbers on the vertical and horizontal axes indicate the grid point count.

Unlike the DAC model which directly inputs the raw SSH from HYCOM, in the WELL model the SSH is spatially compressed using a DWT. As shown in Section 2, the SSH resolution is reduced by a factor of 2 at each compression level, which is accompanied by a loss of information at the small scale as shown in Figure 4. However, the variance of the SSH is increased in the compression process as shown in Figure 5. The effect is particularly visible in the separation phase of the LC system when the frontal eddies of the LC intensify (Chérubin et al., 2005; Donohue et al., 2016). This is due to the preservation of the SSH variance when the size of the grid cell is increased due to the reduction of the grid resolution. Therefore the SSH variance per unit area remains the same. While the SSH patterns remain unchanged during the compression process, so are the EOF modes as shown in Figure 6. The first 6 EOF modes structure is identical between the EOF calculated from the HYCOM and the compressed SSH. The only difference is the magnitude of the modes, which is higher in the compressed SSH modes as a result of the compression process as previously explained. The decompression of the predicted SSH is shown to return realistic SSH levels up to compression level 3, after which the reconstruction becomes very noisy (Figure 7).

### 3.2. Model sensitivity to DWT levels

Because of the information loss associated with the DWT data compression, the performance of the LSTM model vs. the number of DWT levels was evaluated. With more levels, DWT yields a higher compression rate, which may result in the loss of relevant dynamical information. This loss can be observed in the SSH spectral changes associated with the DWT levels (Figure 4). Increasing the DWT level leads to a loss of features in the mesoscale (up to 50 km at the fifth level). These scales are, however less than the dominant wavelengths of the perturbations observed by Donohue et al. (2016) that propagate around the northern front of the LC.

**Figure 8:**
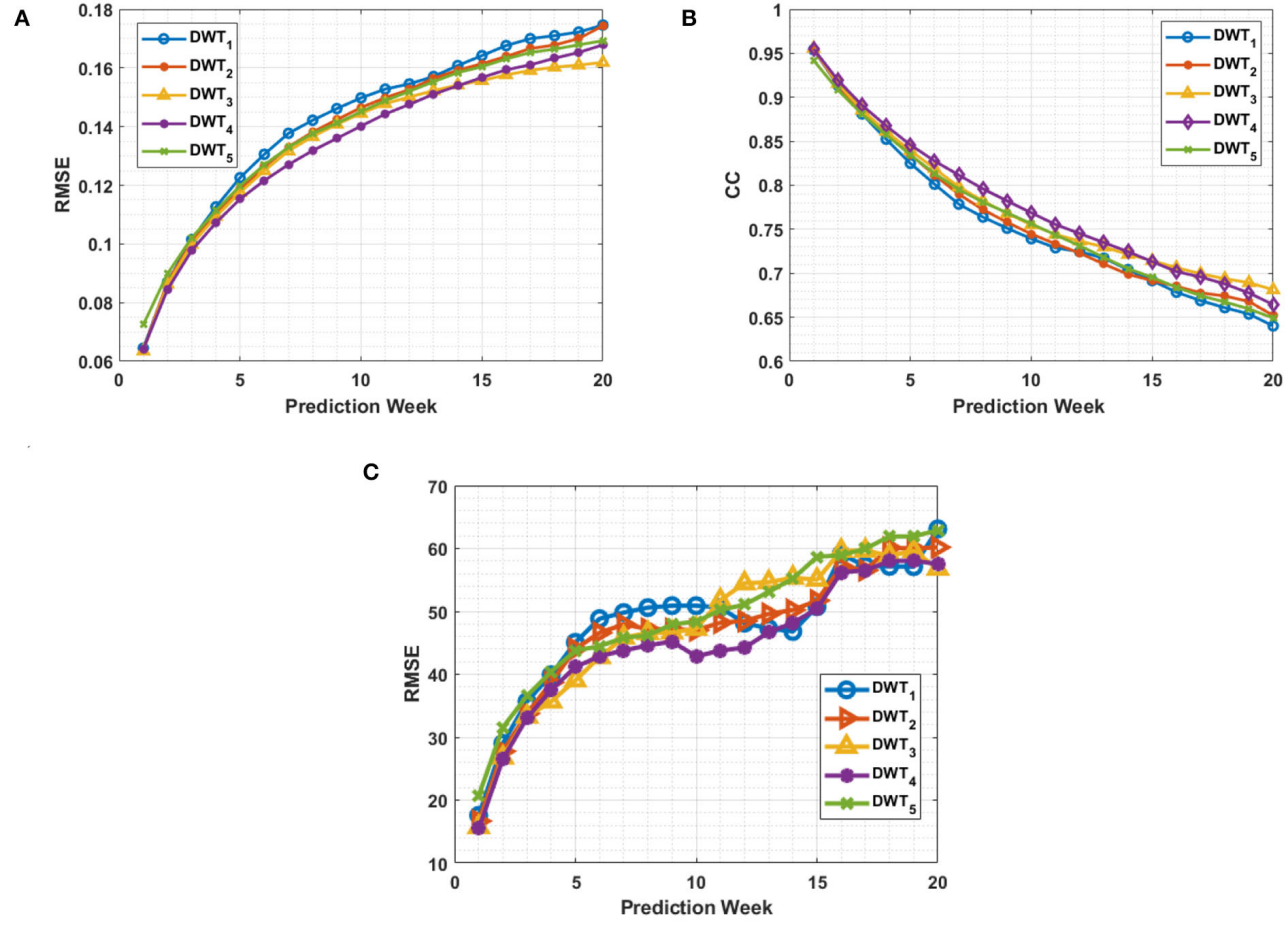
Prediction model performance evaluation of five DWT compression levels using the SSH data from March 2008 to December 2009. **(A)** RMSE. **(B)** Correlation Coefficients. **(C)** Frontal position *FP*_*RMSE*_ in km.

The model performance metrics were calculated over the entire ROI region and their evolution over a 20-week prediction window is shown in Figure 8. Both the CCs and RMSE were slightly improved as the number of levels increased up to 4 and then degraded with a compression level of 5. This implies that the relevant dynamical information of the SSH is effectively selected by the DWT transformation, which slightly improves the SSH prediction with the DWT level. However, because the Details part is not predicted, the transformation back to the original SSH resolution is conducted by replacing the Details with zeros. SSH reconstructed from the 4th and 5th DWT levels show an increase in the SSH noise induced by the zeros of the Details, which is unrealistic (Figure 7). Level 3 DWT decomposition led to the best overall SSH field where the effects of zeros were negligible in the reconstruction. The third level of DWT compression was then used in the rest of this study.

### 3.3. Overall model performance for 20-week long predictions of the loop current SSH

**Figure 9:**
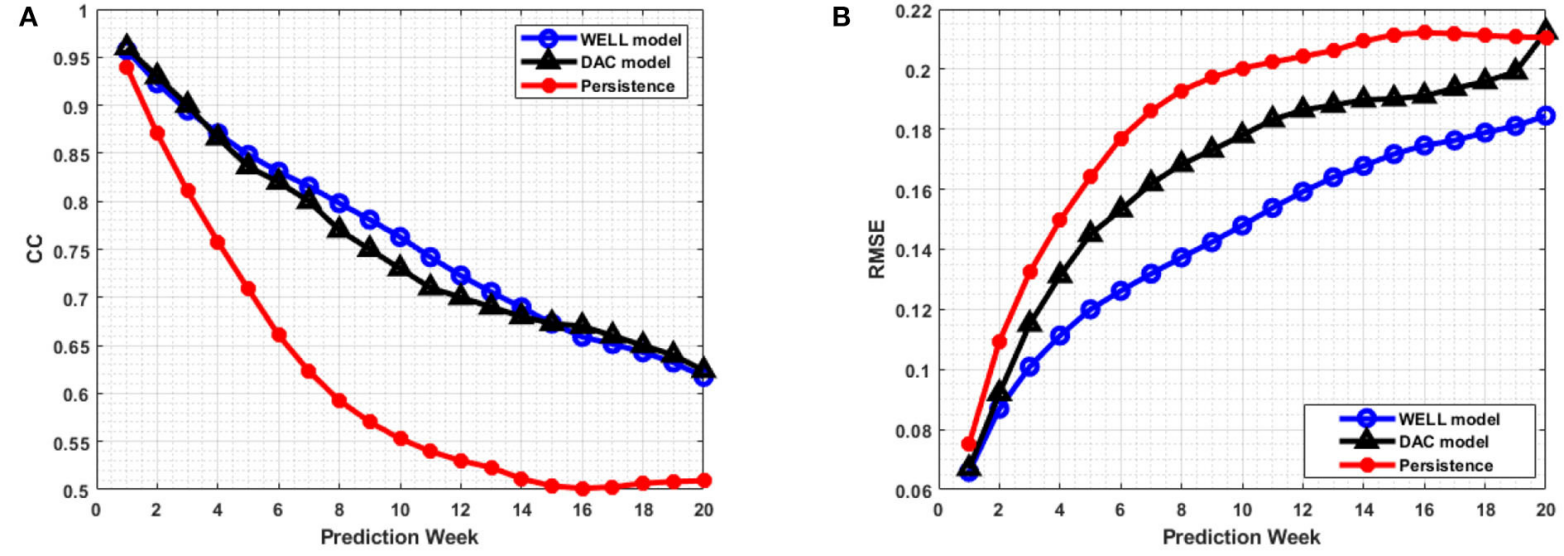
**(A)** Average prediction correlation coefficients (CCs) and **(B)** average prediction RMSEs per week for all 20-week prediction sliding windows over March 2008 to December 2009 period. The horizontal axis is the number of weeks.

The average RMSEs and CCs between the predicted and observed SSH were computed for the defined ROI over March 2008 to December 2009 period and averaged over all 20-week prediction sliding windows (Figure 9). SSH persistence was defined as the first state of the SSH prediction period. The RMSE and CC indicated that the model prediction was better than persistence. However, both performance measures tended toward a plateau, as the model errors did not increase significantly toward the end of the 20-week period. This is because the LC and its eddies remained in the ROI for most of the 20-week period. Overall, the DWT compression did not have much effect on the temporal phase of the feature predicted as shown by the closeness of the CC values between the prediction by the WELL and the DAC model.

**Figure 10:**
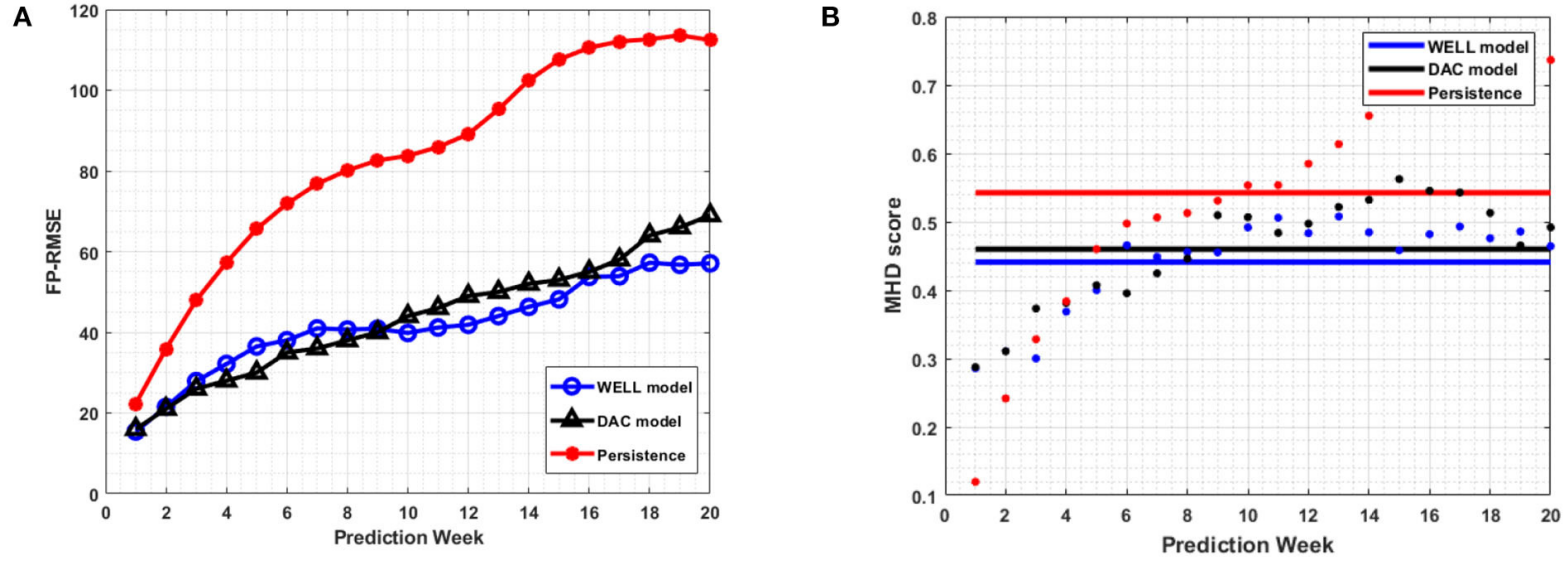
The average *FP*_*RMSE*_ **(A)** and the average MHD score **(B)** per week for all 20-week prediction sliding windows over March 2008 to December 2009 period. The horizontal axis is the number of weeks. The horizontal lines show the mean value of the MHD score for each model and persistence.

The correlation coefficient remained well above 0.6 over the 20-week period, which is well above the 4 to 6-week period of current state-of-the-art data assimilated ocean numerical models (Lin et al., 2007; Yin and Oey, 2007; Xu et al., 2013). However, the WELL model improved the RMSEs by up to 20% at 8 weeks and 11% at 18 weeks over the DAC model (Figure 9B). The frontal position errors of the WELL model were smaller than those of persistence, as shown in Figure 10A. The prediction error of the WELL model was less than in the DAC model after 9 weeks, about 42 km at 12 weeks, less than the 50 km obtained by the DAC model. The frontal position error for the neural network model of Zeng et al. (2015) was at best 60 km at 6 weeks. The DAC model was better for predictions less than 6 weeks ahead with a slight improvement of 5km at best.

The MHD score (Figure 10B) confirmed the overall better agreement of the predicted SSH contours by the WELL model with the observed ones. With this measure (lower scores indicate higher contours similarity), both the DAC model and the WELL model outperformed persistence. The WELL model predicted SSH contours were more similar to the HYCOM SSH most of the weeks than those of the DAC model.

### 3.4. Long-term prediction of Eddy Cameron and Eddy Darwin SSH evolution

**Figure 11:**
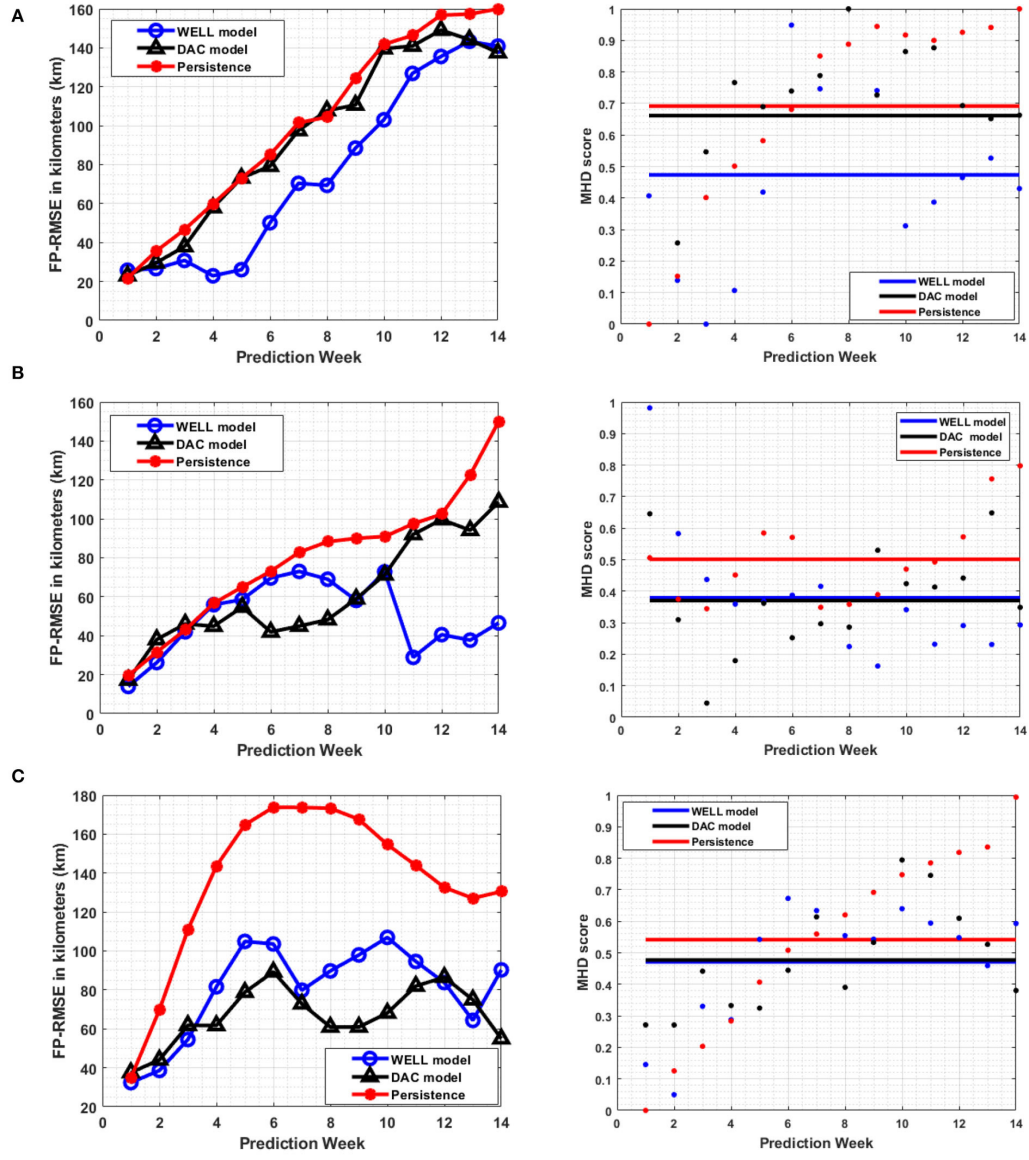
The average *FP*_*RMSE*_ (left column) and the average MHD score (right column) per week for 14-week prediction windows over a four-week sliding window using the 0.45 m contour level of the SSH. **(A)** Eddy Cameron formation in July 2008. **(B)** Eddy Darwin formation in December 2008. **(C)** Loop current leap state in May 2009 (average of 4 weeks). The horizontal lines on the graph in the right column indicate the mean value of the MHD.

During the second half of 2008 and 2009, two major LC eddies were formed: Eddy Cameron (Jul 08 – May 09) and Eddy Darwin (Dec 08 – Nov 09). Both eddies detached and re-attached at least once before separation occurred. The prediction performance for each eddy as measured by the *FP*_*RMSE*_ averaged over 4-week (instead of 1 week in order to capture more changes in the evolution of the eddies) sliding 14-week prediction periods reveals that the WELL model expanded by about 2 weeks the prediction window at a given *FP*_*RMSE*_ over the DAC model for Eddy Cameron. Figure 11A shows that the WELL model's frontal position error is less than 30 km up to 5 weeks ahead, which is much less than the 70 km of the DAC model. The average MHD scores confirm this improvement.

For eddy Darwin (Figure 11B), both models exhibited similar frontal position errors until week 5. The DAC model's frontal position error was lower than the WELL model's until week 9. From week 11 onward, the WELL model frontal position errors exhibited the same values as weeks 2-4 (< 30 km), while the DAC model frontal position errors were above 80 km. The MHD scores also show that the model performances were on average similar, although the similarity of the WELL model was higher than for the DAC model from week 8 onward.

The LC SSH after eddy shedding was also predicted and its performance metrics are shown in Figure 11C. It is characterized by little changes in the dynamics of the LC, concentrated mostly in the southeast region of the GoM for a long period of time (till May 2009) and known as the leap state. Interestingly, the DAC model predictions frontal position errors were similar to or lower than the WELL model's, but the MHD scores were lower for the latter from weeks 10–13. The DAC and the WELL models performed differently for each eddy although both models had more errors predicting eddy Cameron than eddy Darwin as shown by the lower average MHD score for eddy Darwin.

### 3.5. Principal components prediction

**Figure 12:**
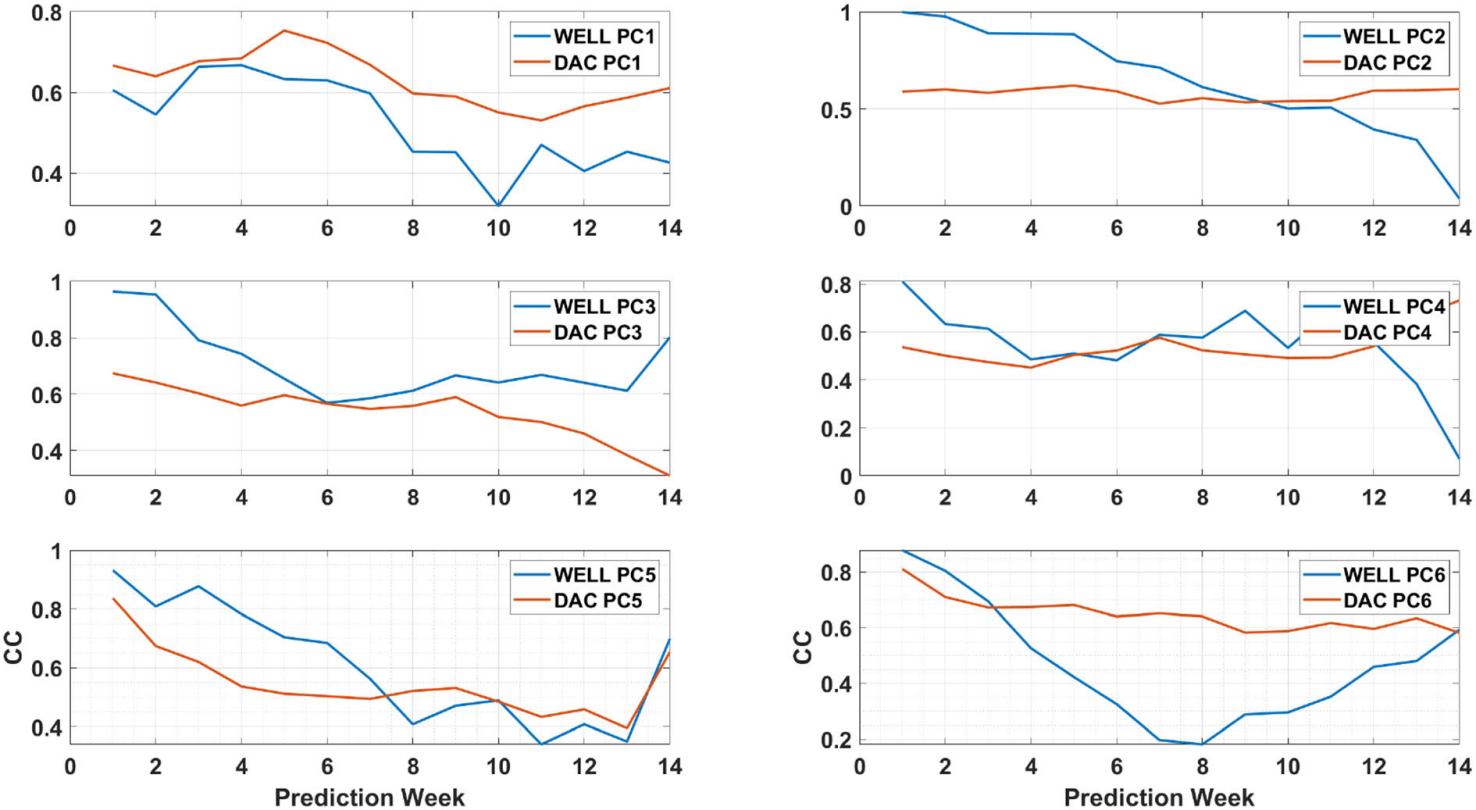
The first six principal components (PCs) linear correlation coefficient (CC) time evolution over a 14-week prediction period of formation of eddy Cameron. The blue (red) solid line shows the WELL (DAC) model PC CCs. For the DAC model, each PC was averaged across all six partitions. The linear correlation coefficients were calculated with HYCOM PCs for the same period.

The EOF analysis is often used to determine the dominant modes of variability of a time series in a statistical manner. The decomposition contains both the spatial patterns (Figure 6) and their weight in the signal variance which is provided by the time varying PCs (Figure 12). The PCs evolution is the output of the LSTM model. The prediction model skills can therefore be assessed through the extent of the differences with the PCs of the HYCOM SSH as done in Wang et al. (2019), while the spatial modes remain the same throughout the prediction process. To do so, we calculated the linear correlation coefficients (CC-Jolliff et al., 2009) for each prediction week of the first 6 PCs with the HYCOM PCs.

The linear CCs time series are shown in Figure 12 for a 14-week long prediction window that encompasses the formation of eddy Cameron. Wang et al. (2019) showed that the two most relevant PCs for the prediction of eddy separation in this dataset (HYCOM) are the PCs associated with EOF modes 2 and 3, called PC2 and PC3, respectively. Figure 12 shows that the correlation for the WELL model is well above 0.6 for PC3 throughout the 14-week prediction window and above 0.5 until week 11 for PC2. For the DAC model, the CC for PC3 is below 0.6 after week 3 but remains almost constant for PC2 and above 0.5. It was less than the PC2 correlation of the WELL model until week 9. As shown in Figure 10, the WELL model frontal position and contour similarity skills were higher than the DAC model skills, which confirms the role of PC3 in the LC evolution.

**Figure 13:**
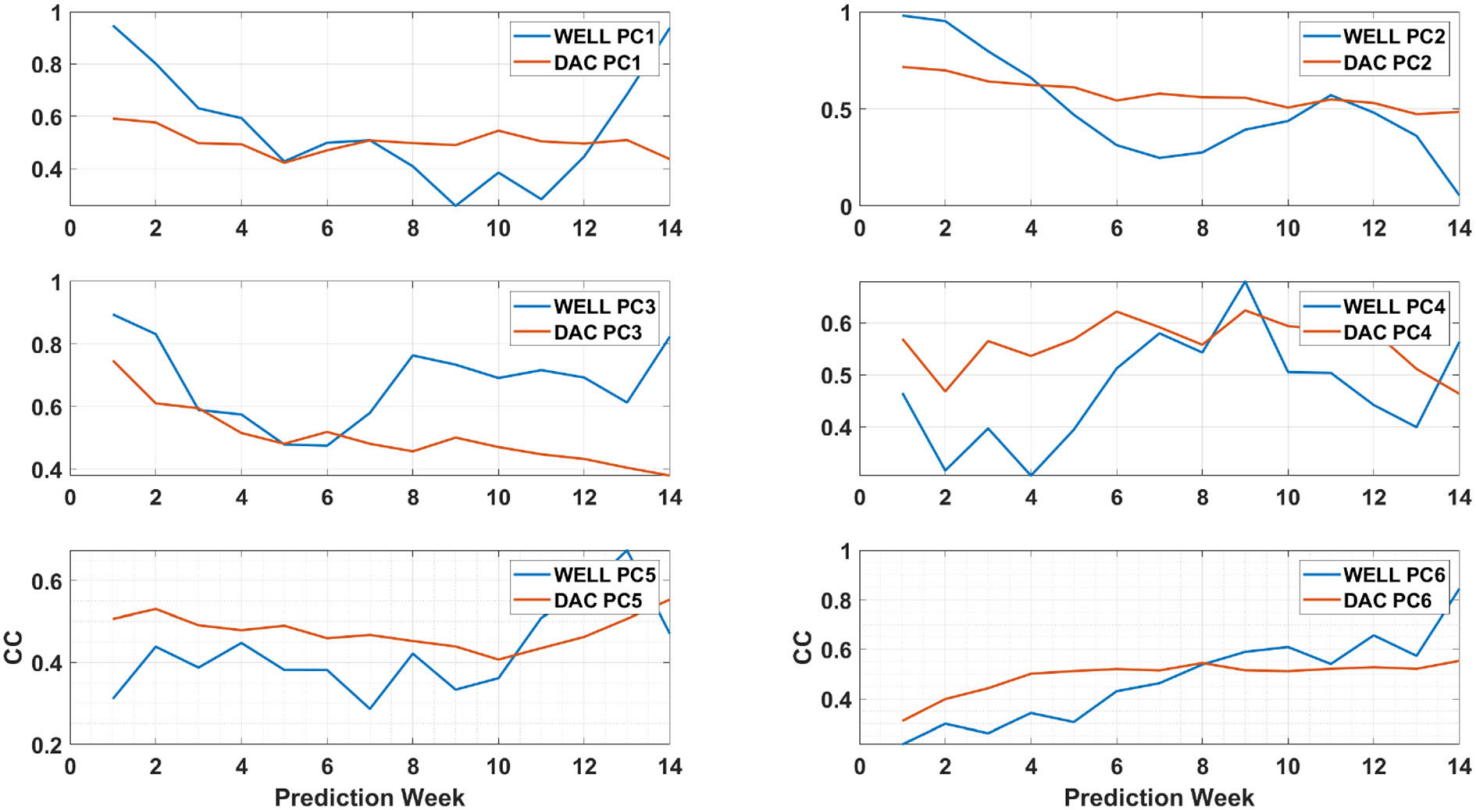
Same as Figure 12 for eddy Darwin.

For Eddy Darwin, the CC evolution of each PC differs from the one of Eddy Cameron (Figure 13). As for Eddy Cameron, PC3 of the WELL model is better correlated with the HYCOM's PC3 than the PC3 of the DAC model. The CC of the WELL model's PC1, PC3, PC5, and PC6 show an increase above the same DAC model's PCs CC values toward the end of the prediction period, starting as early as week 6 prediction for PC3. This difference is reflected by improved frontal position skills of the DAC model until week 10 and of the WELL model after that (Figure 11B). The decrease in the CC of the WELL model's PC2 from weeks 4 to 11 seems to correspond to the lower frontal position skill of the WELL model during that period. As previously shown by Wang et al. (2019), the prediction skills of the LSTM model are achieved through the prediction of the PCs of the most relevant modes, which confirms the role of PC3 and PC2 in the evolution of both eddies.

### 3.6. SSH contours prediction of eddies Cameron and Darwin

**Figure 14:**
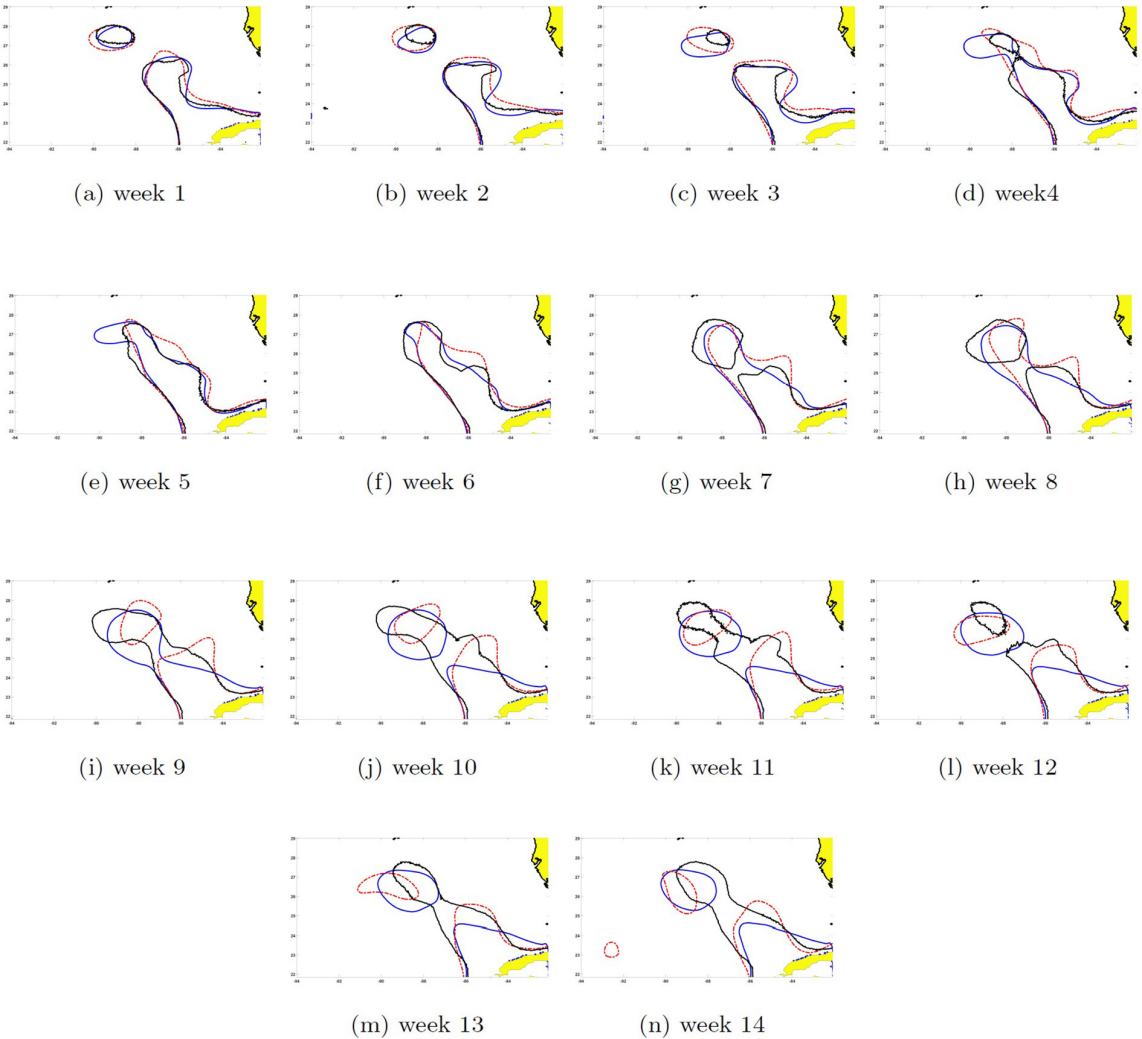
Seas surface height contour (0.45 m) 14-week prediction of eddy Cameron started the third week of May 2008 for the DAC (black solid line) and the WELL (blue solid line) models. The solid red line shows the HYCOM SSH. Week 1 shows the prediction for the first week after the start date. **(a–n)** Week 1–14.

**Figure 15:**
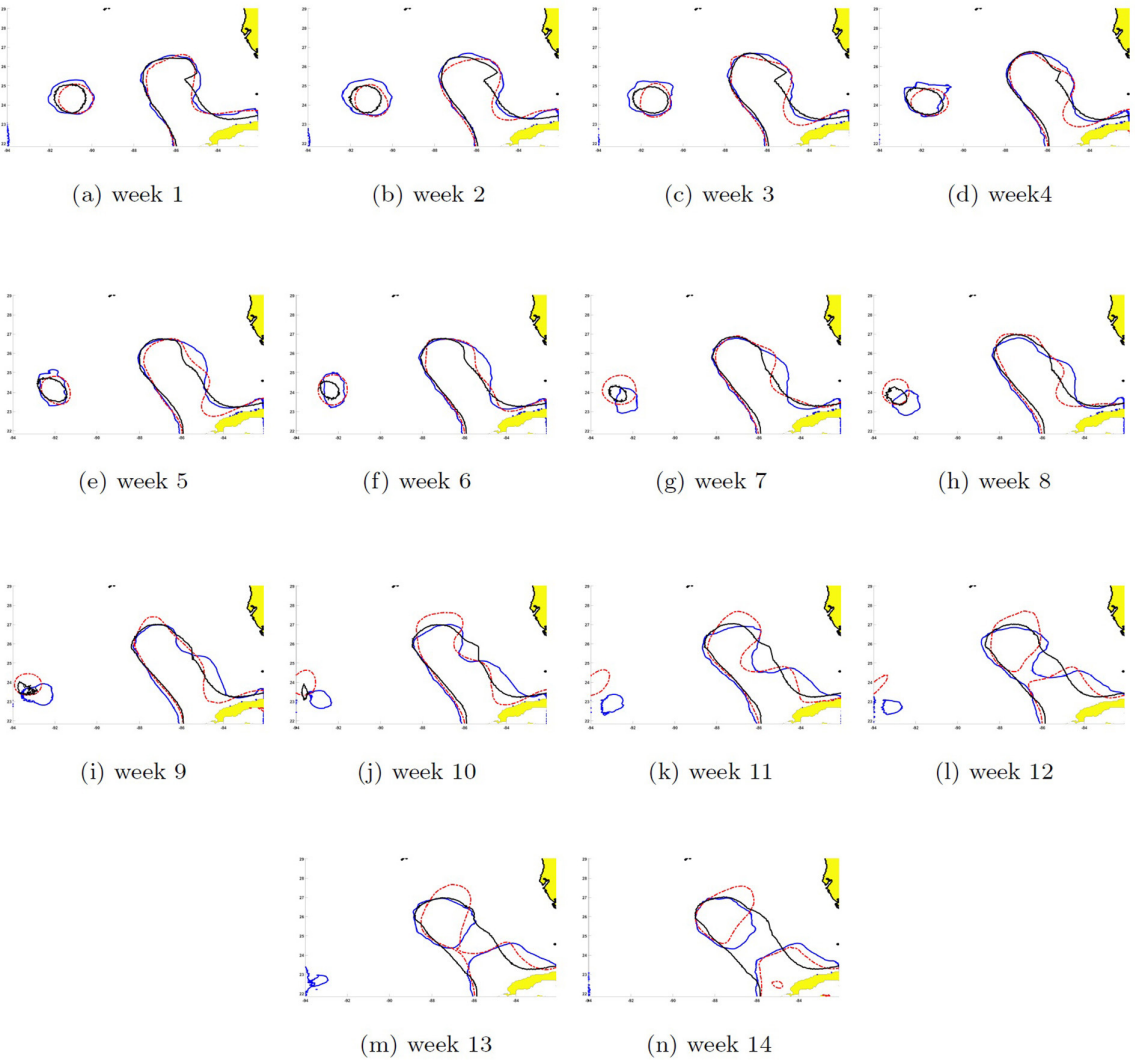
Same as Figure 14 for eddy Darwin. The prediction was started the second week of October 2009. **(a–n)** Week 1–14.

The SSH can be reconstructed by recombining the EOFs with their respective PCs. The dynamics of the shedding process were different between eddies and it is reflected in the contour pattern errors between the two models' prediction and HYCOM SSH shown in Figures 14, 15. The 14-week prediction of eddy Cameron's SSH reveals a better agreement between the WELL model and HYCOM than the DAC model in the critical stages of the LC dynamics. For example, on week 9, the WELL model achieved the separation of the LC eddy at the same time as in HYCOM and kept it separated, unlike the DAC model in which shedding occurred on week 7 (Figure 14). The 14-week prediction of eddy Darwin shows more consistency between all three models through the first 11 weeks (Figure 15). The WELL model appears to better follow the SSH contour oscillations driven by the growth of the baroclinic instability (Yang et al., 2020) during the separation process. Temporary separation occurs on week 12 in HYCOM but in the WELL prediction, the LC is in a necking down position. A change in SSH contour would then show the LCE attached to the LC in the HYCOM model. Full separation occurs on week 14 and is predicted by the WELL model.

## 4. Conclusion

Discrete Wavelet Transform is known to be one of the best compression techniques, especially in image processing (Nashat and Hussain Hassan, 2016). It provides a mathematical tool for encoding information in such a way that it is layered according to the level of detail. The aim of an image compression technique is to reduce the redundancy of the image data in order to improve analysis efficiency. In addition, a wavelet transform fundamentally isolates each frequency in a given signal that may exist at different time resolutions. Transformation of the signal is done by scaling (dilation) and transformation (shifting) functions, which are derived from a mother wavelet. Therefore, DWT acts as a filter that emphasizes frequencies that are most significant to the signal being processed. This concept was applied to two-dimensional simulated SSH data of the GoM in order to predict the evolution of the LC according to the reference model HYCOM. Several levels of compression were tested and resulted in a loss of information at the mesoscale although the scales removed were less relevant to the LC dynamics. However, the benefit of the compression levels was hindered by the reconstruction of the SSH at the original resolution. Indeed the Detail component of the DWT decomposition was not predicted, hence replaced by zeros during the reconstruction process, which added noise to the SSH solution. This method is used by default in the MATLAB Wavelet Toolbox.

Despite the level-3 compression of the original SSH, the WELL model prediction skills were similar if not better than the DAC model, which relied on sub-domain partitions to predict the same area at the same horizontal resolution. The WELL model predicted SSH contour had greater similarity than those of the DAC model. The frontal position error was less than 30 km after 11 weeks for eddy Darwin's prediction. The prediction of PCs revealed that both models showed differences in the prediction of PC3 and PC2, the two most relevant PCs for eddy separation. Correlation with PC3 and PC2 of the HYCOM model was higher with the WELL model PCs than with the DAC model PCs. For the long term prediction of eddy Darwin, the correlation values increased with the length of the prediction. Tracking the 0.45 m SSH contour, the WELL model could predict the final separation of eddy Cameron 10 weeks in advance. For eddy Darwin's separation prediction, the WELL model predicted SSH contour oscillations followed closely by the ones of HYCOM SSH, and the full separation was predicted 14 weeks ahead. It shows that the DWT compression does not negatively affect the SSH prediction and can in fact improve it. This study demonstrates that a similar compression can be applied to the high-resolution prediction of other dynamically active regions of the ocean, which can present a numerical challenge without compression.

Despite the lack of physics in the prediction process which is fully data driven, this LTSM model, trained with long-term simulated SSH data, can achieve long-term predictions that largely surpass the current state-of-the-art ocean numerical models, although not tested with real SSH data. Our model can predict the location of the LC system fronts with an accuracy of 30 km more than 11 weeks in advance, which is unheard of in the realm of numerical ocean model forecasting. Notwithstanding, the biggest limitation to the generalization of the application of this model to real ocean variables, is the data density. Long-term, high-resolution, synoptically consistent time-series are needed that encompass sufficient variability of the dynamical system in order to capture all possible events. Only then the deep learning model can achieve significant long term predictions, that remain out of reach for our current statistical or mathematical tools.

## Data availability statement

Publicly available datasets were analyzed in this study. This data can be found at: https://www.hycom.org/data/goml0pt04/expt-02pt2.

## Author contributions

AM, HZ, and LC conceived and designed the experiments and wrote the paper. AM and AI analyzed the data and performed the experiments. JW did a literature survey and helped develop the prediction model. All authors contributed to the article and approved the submitted version.

## Funding

This study was supported by a grant from the National Academy of Science/United States (NAS/GRP 2000011052)/Understanding Gulf Ocean Systems II to LC and HZ.

## Conflict of interest

The authors declare that the research was conducted in the absence of any commercial or financial relationships that could be construed as a potential conflict of interest.

## Publisher's note

All claims expressed in this article are solely those of the authors and do not necessarily represent those of their affiliated organizations, or those of the publisher, the editors and the reviewers. Any product that may be evaluated in this article, or claim that may be made by its manufacturer, is not guaranteed or endorsed by the publisher.
